# Age at Appendectomy, Pneumonia/Influenza Risk, and The Role of Modifiable Risk-factor Profiles

**DOI:** 10.1007/s44197-026-00612-0

**Published:** 2026-07-11

**Authors:** Gang Chen, Wen Li, Yuhao Zhao, Haitao Li, Yi Li, Chao Song, Pinhua Pan

**Affiliations:** 1 Department of Respiratory Medicine, National Key Clinical Specialty, Branch of National Clinical Research Center for Respiratory Disease, Xiangya Hospital, Central South University, Xiangya Road No 87, Kaifu District, Changsha, Hunan Province 410008 China; 2 Center of Respiratory Medicine, Xiangya Hospital, Central South University, Changsha, 410008 China; 3 Hunan Engineering Research Center for Intelligent Diagnosis and Treatment of Respiratory Disease, Changsha, 410008 China; 4 Nosocomial Infection Control Center, Xiangya Hospital, Central South University, Xiangya Road No 87, Kaifu District, Changsha, Hunan Province 410008 China; 5 National Clinical Research Center for Geriatric Disorders, Xiangya Hospital, Changsha, 410008 China; 6 First Department of Thoracic Medicine, Hunan Cancer Hospital/The Affiliated Cancer Hospital of Xiangya School of Medicine, Central South University, Changsha, 410008 China

**Keywords:** Appendectomy, Pneumonia, Influenza, Modifiable risk-factor profile, Age at appendectomy, Public health

## Abstract

**Background:**

This study examined whether appendectomy was associated with the subsequent risk of pneumonia/influenza and whether this association differed according to age at appendectomy. We also investigated whether modifiable risk-factor profiles were associated with variation in this observed association.

**Methods:**

We conducted a prospective cohort study involving 404,577 participants from the UK Biobank. Participants with appendectomy were categorized into four groups according to age at surgery to evaluate age-specific associations. A weighted modifiable risk-factor score was constructed by summing six risk factors weighted by their corresponding regression coefficients, including smoking status, alcohol consumption, body mass index (BMI), ambient air pollution, physical activity, and diet. Participants with appendectomy were further categorized into quartiles of this score to assess whether the association between appendectomy and pneumonia/influenza differed across modifiable risk-factor profiles.

**Results:**

During a median follow-up period of 13.8 years, a total of 27,589 pneumonia/influenza events were observed. Appendectomy was associated with a higher subsequent risk of pneumonia/influenza (hazard ratio [HR]: 1.10, 95% confidence interval [CI]: 1.06–1.14). In the age-stratified analysis, compared with non-appendectomy participants, appendectomy performed at ages 10–20, 20–30, and ≥ 30 years was associated with higher risks of pneumonia/influenza, with HRs of 1.10 (95% CI: 1.04–1.15), 1.14 (95% CI: 1.06–1.22), and 1.14 (95% CI: 1.06–1.22), respectively, whereas no statistically significant association was observed for appendectomy performed before age 10 (HR: 1.01, 95% CI: 0.92–1.10). Among appendectomy participants, a more favorable modifiable risk-factor profile was associated with a lower risk of pneumonia/influenza. Participants in the lowest quartile of the weighted modifiable risk-factor score had a lower risk than participants without appendectomy (HR: 0.89, 95% CI: 0.82–0.96). These associations were consistent after propensity score matching (PSM).

**Conclusion:**

Appendectomy was associated with a higher subsequent risk of pneumonia/influenza, particularly when performed after childhood. A favorable modifiable risk-factor profile was associated with attenuation of this observed association. From a public health perspective, appendectomy history and age at surgery may help identify individuals who could benefit from targeted respiratory infection prevention strategies, including smoking cessation, weight management, physical activity promotion, dietary improvement, promotion of vaccination, and reduction of environmental exposure.

**Supplementary Information:**

The online version contains supplementary material available at https://doi.org/10.1007/s44197-026-00612-0.

## Introduction

Pneumonia and influenza are critical global infectious diseases responsible for substantial morbidity and mortality each year, posing serious threats to public health and leading to considerable economic burdens worldwide [1, 2]. Despite extensive vaccination efforts, approximately one billion people worldwide still experience symptomatic influenza each year, with severe cases comprising 0.3% to 0.5%, and 25 out of every 10,000 adults are hospitalized with pneumonia annually [3, 4]. The in-hospital mortality rate for community-acquired pneumonia ranges from 4% to 18%, while hospital-acquired pneumonia can result in mortality rates as high as 20% to 30%; children and elderly patients with pneumonia are at especially high risk of mortality [5, 6]. Currently, pneumonia and influenza have emerged as major causes of infectious disease-related mortality [7], making effective disease prevention efforts particularly important.

Previous studies have indicated that gastrointestinal disorders, such as gastroesophageal reflux disease and inflammatory bowel disease, are associated with the development of pneumonia/influenza [8, 9]. Appendicitis is a common gastrointestinal condition and surgical removal is the primary treatment [10]. However, the appendix functions as an immune organ and plays a role in maintaining immune homeostasis as well as the stability and diversity of the gastrointestinal microbiota [11–13]. Therefore, appendectomy may have subtle and long-term associations with host immune and microbial homeostasis. Considering the immunoregulatory role of the appendix and its potential interactions with pulmonary immunity via the gut-lung axis, we hypothesize that appendectomy may be associated with subsequent risk of pneumonia/influenza. Previous studies suggest that the timing of appendectomy across the lifespan may be associated with different health outcomes [14, 15]. Therefore, it is worth further exploring whether undergoing appendectomy at different ages is linked to differences in the subsequent risk of pneumonia or influenza. Several additional risk factors–such as smoking, alcohol consumption, and air pollution–may also contribute to the development of pneumonia and influenza through distinct pathogenic mechanisms [16–18]. Prior studies suggest that favorable lifestyle patterns and effective management of modifiable risk factors are associated with lower risks of certain diseases, potentially through pathways involving inflammation and immunity [19–21], while it remains unclear whether modifiable risk-factor profiles may influence the association between appendectomy and subsequent pneumonia/influenza risk.

This study aims to systematically investigate the prospective association between appendectomy and incident pneumonia/influenza based on a large-scale cohort from UK Biobank. In addition, by comparing the risk of pneumonia/influenza among participants with different modifiable risk-factor profiles, we further evaluated whether the observed association between appendectomy and pneumonia/influenza differed across modifiable risk-factor profiles. These findings may offer significant implications for optimizing preventive strategies and improving clinical management.

## Methods

### Study Population

The UK Biobank is a prospective population-based cohort comprising approximately 500,000 individuals recruited between 2006 and 2010 from 22 assessment centers across England, Wales, and Scotland. Participants underwent comprehensive baseline evaluations, including sociodemographic surveys, lifestyle assessments, physical measurements, and biospecimen collection. Longitudinal health outcomes were ascertained through linkage to national electronic health records, encompassing primary care, hospital admissions, cancer registries, and death certifications, with validated data completeness and accuracy. Detailed descriptions of the study design and data collection procedures have been published elsewhere [22].

### Ethics Statement

The study protocol received ethical approval from the North West Multicenter Research Ethics Committee (11/NW/0382), and all participants provided informed written consent. The inclusion and exclusion process of this study is shown in Supplementary Fig. 1.

### Identification of Exposure

The exposure in this study was appendectomy. We defined participants’ history of appendectomy using data from Operative Procedures-3 (codes 441, 443, 444), Operative Procedures-4 (codes H01 and H02), and operation codes (code 1458), and recorded their age at the time of surgery (Supplementary Fig. 2).

### Identification of Outcomes

The outcomes were defined as the first occurrence of pneumonia or influenza, with a diagnosis of either condition considered as the endpoint event. Pneumonia included viral, bacterial, and other pathogen-induced types. Influenza included both identified viral influenza and unidentified viral influenza [2, 23]. The diagnosis of pneumonia and influenza was based on the International Classification of Diseases codes of the 10th revision (J09-J18). The follow-up period was defined as the time from the baseline assessment date to the date of pneumonia or influenza occurrence, loss to follow-up, the date of participant death, or the end of follow-up on March 1, 2023, whichever occurred first. Participants were excluded if the outcomes occurred before baseline recruitment or prior to the appendectomy procedure.

### Air Pollution Score and Weighted Modifiable Risk-factor Score

According to previous studies and guidelines [16–18], we selected six modifiable risk determinants related to pneumonia and influenza: smoking status, alcohol consumption, body mass index (BMI), ambient air pollution, physical activity and diet to characterize modifiable risk-factor profiles. Ambient air pollutants included PM_2.5_, PM_10_, PM_coarse_, NO_2_, and NO_x_. Annual average concentrations of these pollutants were estimated using land-use regression models developed by the European Study of Cohorts for Air Pollution Effects (ESCAPE) [24, 25]. Each pollutant was included as an independent variable in the regression analysis for outcomes to obtain its respective β coefficient (Supplementary Table 1). We constructed a weighted air pollution score by summing concentrations of the five air pollutants, each weighted by its corresponding β coefficient (Supplementary Fig. 3). The equation was: air pollution score = (β [PM_2.5_] * PM_2.5_ + β [PM_10_] * PM_10_ + β [PM_coarse_] * PM_coarse_ + β [NO_2_] * NO_2_ + β [NO_x_] * NO_x_) / (sum of the β coefficients). A higher air pollution score indicated greater exposure to ambient air pollution. A weighted modifiable risk-factor score was calculated by integrating the air pollutants with the other five risk factors (smoking status, alcohol consumption, BMI, physical activity, diet) in the same weighted manner, with higher scores indicating a less favorable modifiable risk-factor profile and a greater cumulative burden of modifiable risk factors (Supplementary Fig. 4). The equation was: weighted modifiable risk-factor score = (β [smoking status] * smoking status + β [alcohol consumption] * alcohol consumption + β [BMI] * BMI + β [physical activity] * physical activity + β [diet] * diet + β [PM_2.5_] * PM_2.5_ + β [PM_10_] * PM_10_ + β [PM_coarse_] * PM_coarse_ + β [NO_2_] * NO_2_ + β [NO_x_] * NO_x_) / (sum of the β coefficients). Participants were then categorized into quartiles based on their weighted modifiable risk-factor scores. This score was used to characterize modifiable risk-factor profiles and to support stratified association analyses, rather than to develop an individual-level clinical prediction model.

### Other Covariates

Other relevant covariates comprised age, sex, ethnicity, education level, Townsend deprivation index (TDI) and past medical history, including hypertension, diabetes, chronic obstructive pulmonary disease (COPD), asthma, stroke and coronary heart disease (CHD). Demographic information for participants was obtained from baseline data, and detailed information on covariates is provided in Supplementary Table 2.

### Statistical Analyses

Continuous variables were presented as mean ± standard deviation, and categorical variables were presented as frequency and percentage. Differences between groups were assessed using t-tests or chi-square tests. For each appendectomy participant, we employed propensity score matching (PSM) with a caliper of 0.1 to match non-appendectomy participants at a 1:1 ratio based on all covariates, thereby reducing the influence of potential confounding factors on the results. Our study was broadly divided into three parts: 1). Exploring the association between appendectomy and pneumonia/influenza: We employed Cox proportional hazards regression models with follow-up time as the time scale. The strength of the association was estimated using hazard ratios (HRs) and 95% confidence intervals (CIs). Schoenfeld residuals and Kaplan-Meier (KM) curves were used to assess the proportional hazards assumption, and all tests met the pre-specified criteria. Three models were constructed: Model 1 was adjusted for age and sex; Model 2 was adjusted for age, sex, ethnicity, education level, and TDI; Model 3 was further adjusted for BMI, smoking status, alcohol consumption, physical activity, diet, air pollution score, and prior medical history including hypertension, diabetes, COPD, asthma, CHD, and stroke. In subgroup analyses, stratification was performed based on sex (male, female), age (< 60 years, ≥ 60 years), BMI (< 25, 25–30, > 30), smoking status (never/past smokers, current smokers), alcohol consumption (≤ 4 times/week, > 4 times/week), TDI (< mean level, ≥mean level), history of COPD (yes, no) and history of asthma (yes, no). 2). Investigating the association between age at appendectomy and pneumonia/influenza: First, given the developmental changes in immune maturation, the functional importance and age-related involution of appendiceal lymphoid tissue, we divided appendectomy participants into four groups based on their age at surgery: <10, 10–20, 20–30, and ≥ 30 years. This categorization approximately corresponded to childhood, adolescence/early adulthood, young adulthood, and later adulthood, respectively. These cutoffs were selected based on the age-related development and involution of appendiceal lymphoid tissue, as well as life-course changes in systemic immune function. They were also chosen to balance biological interpretability with statistical feasibility by maintaining sufficient sample size and pneumonia/influenza events in each subgroup. Age at appendectomy was treated as a categorical variable to assess whether appendectomy at different life stages was associated with differential risks of developing pneumonia/influenza (Supplementary Table 3). Second, age at appendectomy was analyzed as a continuous variable to examine linear trends, and restricted cubic splines (RCS) were used to investigate potential linear or nonlinear associations between age at appendectomy and the incidence of pneumonia/influenza. In all analyses within this section, participants without appendectomy were used as the reference group, and the three Cox models were adjusted for the same covariates as described above. 3). Evaluating the association between modifiable risk-factor profiles and pneumonia/influenza among participants with appendectomy: Participants with appendectomy were divided into four groups based on quartiles of the weighted modifiable risk-factor score, with non-appendectomy participants serving as the reference group. A fully adjusted Cox model was used to assess the association between different modifiable risk-factor profiles and the incidence of pneumonia/influenza, excluding covariates already incorporated into the weighted modifiable risk-factor score (Supplementary Table 4).

Additionally, five sensitivity analyses were conducted to assess the robustness of the results: (1) excluding participants with self-reported pneumonia or influenza, to minimize potential outcome misclassification due to recall bias and improve diagnostic accuracy; (2) excluding incident cases during the first two years of follow-up to reduce the influence of reverse causation; (3) excluding participants with a history of appendicitis at baseline, to distinguish the association of appendectomy from that of the underlying inflammatory condition; (4) performing multiple imputation for missing covariates, to reduce potential bias introduced by incomplete data and enhance the robustness and validity of the estimates; and (5) restricting the analysis to participants exposed to air pollutant concentrations complying with European Union (EU) limits for PM_2.5_ (25 µg/m^3^), PM_10_ (40 µg/m^3^), and NO_2_ (40 µg/m^3^), to minimize potential distortion of the exposure–outcome association related to extreme environmental exposures. Additional component-specific analyses were performed using pneumonia and influenza as separate outcomes to examine whether the association observed for the composite endpoint was driven primarily by either component. Statistical analyses were performed using R statistical software version 4.1.3 (R Foundation for Statistical Computing, Vienna, Austria), with a significance level of *P* < 0.05.

## Results

### Baseline Characteristics of Included Participants

After excluding participants with pneumonia or influenza at baseline and those with missing covariates, a total of 404,577 participants were included in the final analysis. The baseline characteristics of included participants were presented in Table 1. Participants who underwent appendectomy tended to be older, were more likely to be female, had higher BMI, and were more likely to be current smokers and to report higher alcohol consumption. Additionally, compared with non-appendectomy participants, those in the appendectomy group had a higher prevalence of comorbidities.

**Table 1:** Baseline characteristics of included participants

Characteristics	Total (*N* = 404577)	Before PSM		*P*	After PSM		SMD
		Non-Appendectomy (*N* = 357636)	Appendectomy (*N* = 46941)		Non-Appendectomy (*N* = 46939)	Appendectomy (*N* = 46939)	
Age, mean (SD)	57.0 (8.1)	56.8 (8.1)	58.5 (7.7)	< 0.001	58.5 (7.8)	58.5 (7.7)	0.002
Male, n (%)	184,353 (45.6)	164,382 (46.0)	19,971 (42.5)	< 0.001	20,081 (42.8)	19,970 (42.5)	0.005
Ethnicity, White, n (%)	384,327 (95.0)	338,885 (94.8)	45,442 (96.8)	< 0.001	45,401 (96.7)	45,440 (96.8)	0.005
Education Level, n (%)				< 0.001			0.006
High	195,632 (48.4)	172,086 (48.1)	23,546 (50.2)		23,572 (50.2)	23,544 (50.2)	
Medium	75,145 (18.6)	66,956 (18.7)	8189 (17.4)		8143 (17.3)	8189 (17.4)	
Low	70,646 (17.5)	62,805 (17.6)	7841 (16.7)		7925 (16.9)	7841 (16.7)	
None of the above	63,154 (15.6)	55,789 (15.6)	7365 (15.7)		7299 (15.5)	7365 (15.7)	
BMI category, n (%)				< 0.001			0.013
< 25 kg/m^2^	135,770 (33.6)	120,783 (33.8)	14,987 (31.9)		14,752 (31.4)	14,987 (31.9)	
25–30 kg/m^2^	172,688 (42.7)	152,685 (42.7)	20,003 (42.6)		20,005 (42.6)	20,003 (42.6)	
> 30 kg/m^2^	96,119 (23.8)	84,168 (23.5)	11,951 (25.5)		12,182 (26.0)	11,949 (25.5)	
Townsend deprivation index, mean (SD)	-1.4 (3.0)	-1.4 (3.0)	-1.5 (2.9)	< 0.001	-1.5 (2.9)	-1.5 (2.9)	0.008
Smoking status, n (%)				0.001			0.002
Never or Past	364,368 (90.1)	322,297 (90.1)	42,071 (89.6)		42,044 (89.6)	42,070 (89.6)	
Current	40,209 (9.9)	35,339 (9.9)	4870 (10.4)		4895 (10.4)	4869 (10.4)	
Alcohol consumption, n (%)				< 0.001			0.002
≤ 4 times a week	319,596 (79.0)	282,977 (79.1)	36,619 (78.0)		36,649 (78.1)	36,618 (78.0)	
Daily or almost daily	84,981 (21.0)	74,659 (20.9)	10,322 (22.0)		10,290 (21.9)	10,321 (22.0)	
Physical activity, n (%)	313,706 (77.5)	277,562 (77.6)	36,144 (77.0)	0.003	36,042 (76.8)	36,144 (77.0)	0.005
Healthful diet, n (%)	227,645 (56.3)	200,066 (55.9)	27,579 (58.8)	< 0.001	27,502 (58.6)	27,577 (58.8)	0.003
PM_2.5_, mean (SD)	10.0 (1.1)	10.0 (1.1)	9.9 (1.1)	< 0.001	10.0 (1.1)	9.9 (1.1)	0.012
PM_10_, mean (SD)	19.3 (1.9)	19.3 (1.9)	19.2 (2.0)	0.001	19.2 (1.9)	19.2 (2.0)	0.007
PM_coarse_, mean (SD)	6.4 (0.9)	6.4 (0.9)	6.4 (0.9)	0.424	6.4 (0.9)	6.4 (0.9)	0.011
NO_2_, mean (SD)	29.1 (9.2)	29.1 (9.2)	28.8 (9.3)	< 0.001	28.9 (9.1)	28.8 (9.3)	0.010
NO_x_, mean (SD)	43.7 (15.5)	43.8 (15.5)	43.3 (15.7)	< 0.001	43.6 (15.5)	43.3 (15.7)	0.016
Air pollution score, mean (SD)	13.4 (1.8)	13.4 (1.8)	13.4 (1.9)	< 0.001	13.4 (1.8)	13.4 (1.9)	0.009
Prevalent disease, n (%)							
Hypertension	106,364 (26.3)	92,831 (26.0)	13,533 (28.8)	< 0.001	13,429 (28.6)	13,531 (28.8)	0.005
Diabetes	19,998 (4.9)	17,332 (4.8)	2666 (5.7)	< 0.001	2720 (5.8)	2665 (5.7)	0.005
COPD	5990 (1.5)	5065 (1.4)	925 (2.0)	< 0.001	947 (2.0)	923 (2.0)	0.004
Asthma	44,901 (11.1)	39,190 (11.0)	5711 (12.2)	< 0.001	5760 (12.3)	5709 (12.2)	0.003
CHD	20,276 (5.0)	17,273 (4.8)	3003 (6.4)	< 0.001	3012 (6.4)	3001 (6.4)	0.001
Stroke	5487 (1.4)	4702 (1.3)	785 (1.7)	< 0.001	802 (1.7)	783 (1.7)	0.003

Data were expressed as mean (SD) and count (%). *P* values were computed using the t-test, or Chi-square test, as appropriate. Abbreviations: *PSM*, propensity score matching; *SD*, standard deviation; *SMD*, standardized mean difference; *BMI*, body mass index; *COPD*, chronic obstructive pulmonary disease; *CHD*, coronary heart disease

### Appendectomy and Pneumonia/Influenza Occurrence

**Fig. 1:**
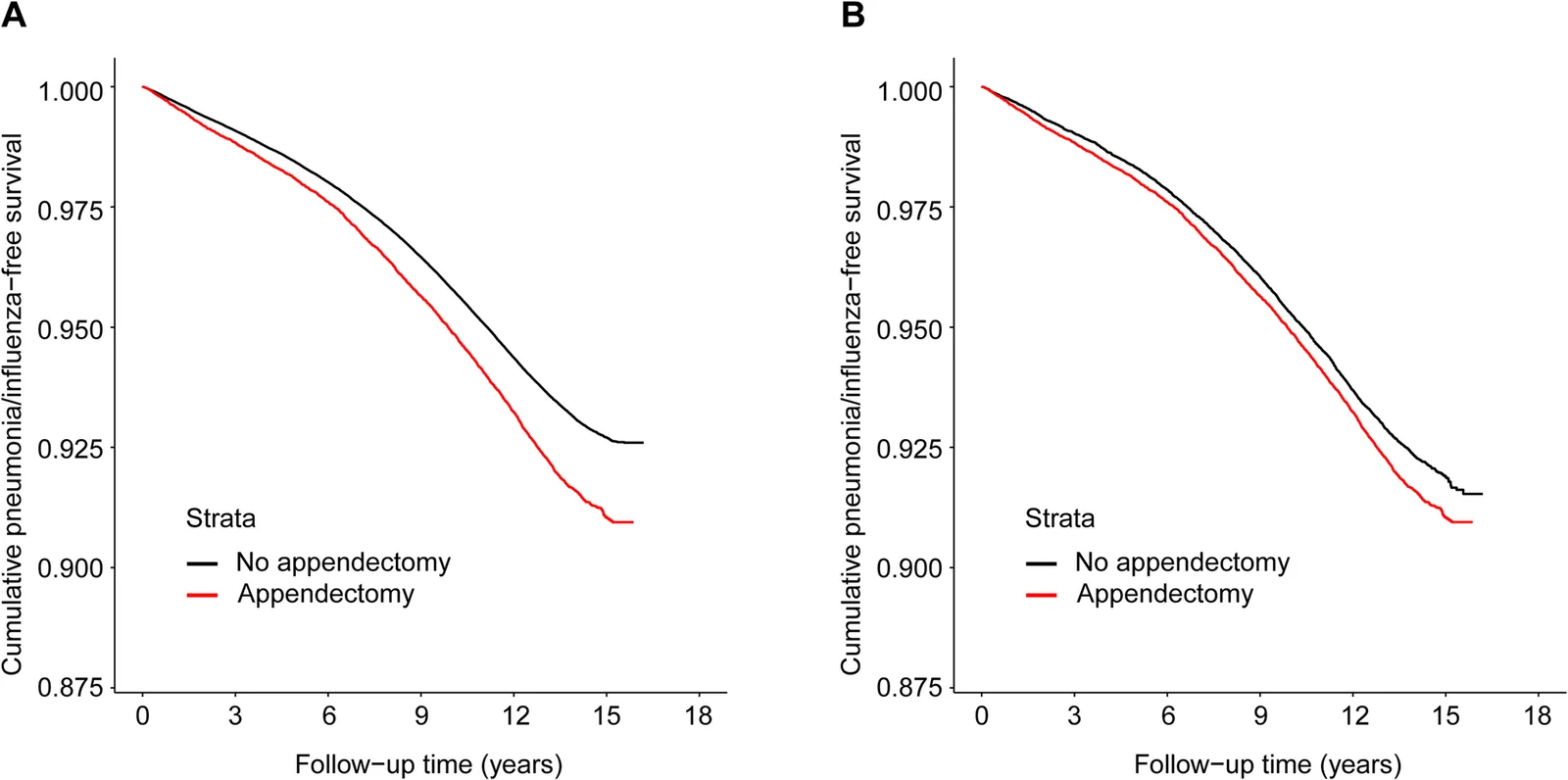
Kaplan-Meier plots for cumulative proportion of surviving participants who did not have pneumonia/influenza during the follow-up in relation to appendectomy. **A**. Before PSM; **B**. After PSM. Abbreviations: PSM, propensity score matching

During a median follow-up period of 13.8 years, a total of 27,589 pneumonia/influenza events were observed. KM curves revealed that participants who underwent appendectomy had a higher incidence rate of pneumonia/influenza during the follow-up period (Fig. 1). Specifically, the incidence was 5.1 per 1,000 person-years in the non-appendectomy group and 5.7 per 1,000 person-years in the appendectomy group. Cox proportional hazards regression models further showed that appendectomy was associated with a higher subsequent risk of pneumonia/influenza. The HRs were 1.13 (95% CI: 1.09–1.17) in Model 1, 1.15 (95% CI: 1.11–1.19) in Model 2 and 1.10 (95% CI: 1.06–1.14) in Model 3. Similar results were observed in the PSM-matched dataset, with HRs of 1.10 (95% CI: 1.05–1.16), 1.10 (95% CI: 1.06–1.16), and 1.11 (95% CI: 1.06–1.16) in the respective models (Table 2).

**Table 2:** Hazard ratios for pneumonia/influenza associated with appendectomy

	Exposure	Event/Total	Incidence (per 1000 person-year)	Model 1 HR (95% CI)	Model 2 HR (95% CI)	Model 3 HR (95% CI)
Before PSM	Non-Appendectomy	23,786/357,636	5.1	1.00	1.00	1.00
	Appendectomy	3803/46,941	5.7	1.13 (1.09–1.17)	1.15 (1.11–1.19)	1.10 (1.06–1.14)
After PSM	Non-Appendectomy	3468/46,939	5.7	1.00	1.00	1.00
	Appendectomy	3802/46,939	6.2	1.10 (1.05–1.16)	1.10 (1.06–1.16)	1.11 (1.06–1.16)

Model 1 was adjusted for age and sex; Model 2 was adjusted for age, sex, ethnicity, Townsend deprivation index, education level; Model 3 was further adjusted for BMI, smoking status, alcohol consumption, physical activity, healthful diet, air pollution score, prevalent diseases (hypertension, diabetes, COPD, asthma, CHD, stroke). Abbreviations: *PSM*, propensity score matching; *HR*, hazard ratio; *CI*, confidence interval; *BMI*, body mass index; *COPD*, chronic obstructive pulmonary disease; *CHD*, coronary heart disease

In subgroup analyses performed using fully adjusted models, potential interactions between appendectomy and sex, as well as between appendectomy and TDI stratification, were identified; no significant interactions were observed between appendectomy and other subgroups (Supplementary Fig. 5).

### Age at Appendectomy and Pneumonia/Influenza Occurrence

**Fig. 2:**
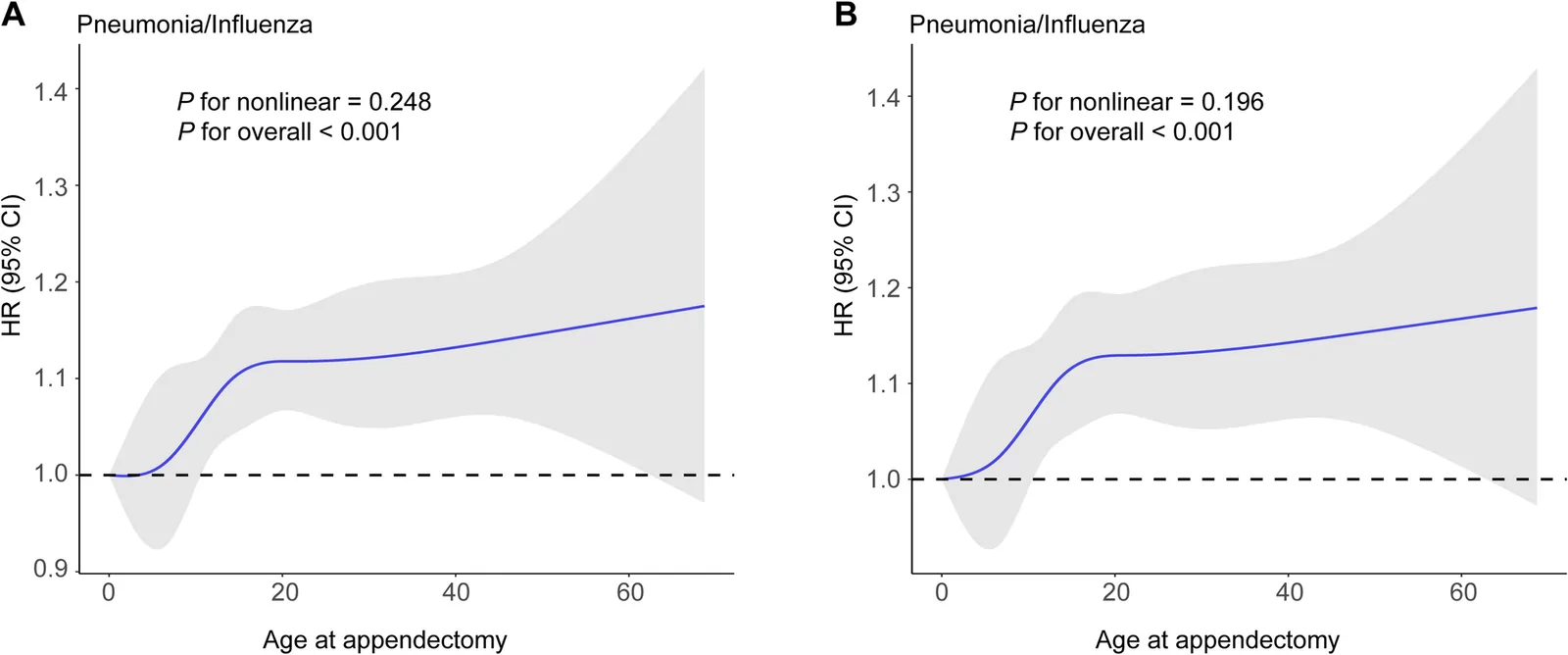
Restricted cubic spline models for the association between age at appendectomy and pneumonia/influenza risk. **A**. Before PSM; **B**. After PSM. Abbreviations: PSM, propensity score matching; HR, hazard ratio; CI, confidence interval

In the stratified analysis by age at appendectomy, we observed that the incidence of pneumonia/influenza per 1000 person-years increased with advancing age at surgery (from 5.1 to 6.0). In Model 3 adjusted for all covariates, participants who underwent appendectomy between the ages of 10 and 20 had a higher incidence of pneumonia/influenza compared with participants without appendectomy (HR: 1.10, 95% CI: 1.04–1.15), while those who had the surgery at ages 20–30 also showed a higher incidence of the outcomes (HR: 1.14, 95% CI: 1.06–1.22). Participants who underwent appendectomy after the age of 30 similarly showed a higher incidence of pneumonia/influenza (HR: 1.14, 95% CI: 1.06–1.22). In the PSM-matched dataset, appendectomy performed at ages 10–20, 20–30, and ≥ 30 years remained associated with higher risks of pneumonia/influenza, with fully adjusted HRs ranging from 1.11 to 1.15 across these age-at-surgery groups. Appendectomy before the age of 10 was not significantly associated with subsequent pneumonia/influenza occurrence, with HRs of 1.01 (95% CI: 0.92–1.10) before PSM and 1.02 (95% CI: 0.92–1.12) after PSM (Table 3). In addition, we modeled age as a continuous exposure using RCS and observed a potential linear relationship between the risk of pneumonia/influenza and age at appendectomy (Fig. 2); higher estimated risks were observed with older age at surgery, and no single inflection point was identified on the spline curve.

**Table 3:** Hazard ratios for pneumonia/influenza associated with appendectomy by age at surgery

Exposure	Before PSM					After PSM				
	Event/Total	Incidence (per 1000 person-year)	Model 1 HR (95% CI)	Model 2 HR (95% CI)	Model 3 HR (95% CI)	Event/Total	Incidence (per 1000 person-year)	Model 1 HR (95% CI)	Model 2 HR (95% CI)	Model 3 HR (95% CI)
Non-Appendectomy	23,786/357,636	5.1	1.00	1.00	1.00	3468/46,939	5.7	1.00	1.00	1.00
Appendectomy at age < 10 years	496/7142	5.1	0.98 (0.90–1.07)	1.02 (0.93–1.12)	1.01 (0.92–1.10)	496/7142	5.6	0.96 (0.87–1.05)	0.98 (0.89–1.08)	1.02 (0.92–1.12)
Appendectomy at age 10–20 years	1724/21,449	5.8	1.14 (1.08–1.19)	1.15 (1.10–1.21)	1.10 (1.04–1.15)	1723/21,447	6.2	1.11 (1.05–1.17)	1.11 (1.04–1.17)	1.11 (1.05–1.17)
Appendectomy at age 20–30 years	829/9611	5.9	1.19 (1.11–1.28)	1.20 (1.12–1.29)	1.14 (1.06–1.22)	829/9611	6.4	1.16 (1.08–1.25)	1.16 (1.07–1.25)	1.15 (1.06–1.24)
Appendectomy at age ≥ 30 years	754/8739	6.0	1.17 (1.09–1.26)	1.18 (1.10–1.27)	1.14 (1.06–1.22)	754/8739	6.5	1.14 (1.06–1.24)	1.14 (1.05–1.23)	1.15 (1.06–1.24)
			*P*_trend_ < 0.001	*P*_trend_ < 0.001	*P*_trend_ < 0.001			*P*_trend_ < 0.001	*P*_trend_ < 0.001	*P*_trend_ < 0.001

Model 1 was adjusted for age and sex; Model 2 was adjusted for age, sex, ethnicity, Townsend deprivation index, education level; Model 3 was further adjusted for BMI, smoking status, alcohol consumption, physical activity, healthful diet, air pollution score, prevalent diseases (hypertension, diabetes, COPD, asthma, CHD, stroke). Abbreviations: *PSM*, propensity score matching; *HR*, hazard ratio; *CI*, confidence interval; *BMI*, body mass index; *COPD*, chronic obstructive pulmonary disease; *CHD*, coronary heart disease. *P*_trend_ was performed by treating the variables continuously

### Modifiable Risk-factor Profiles and Pneumonia/Influenza Occurrence

**Fig. 3:**
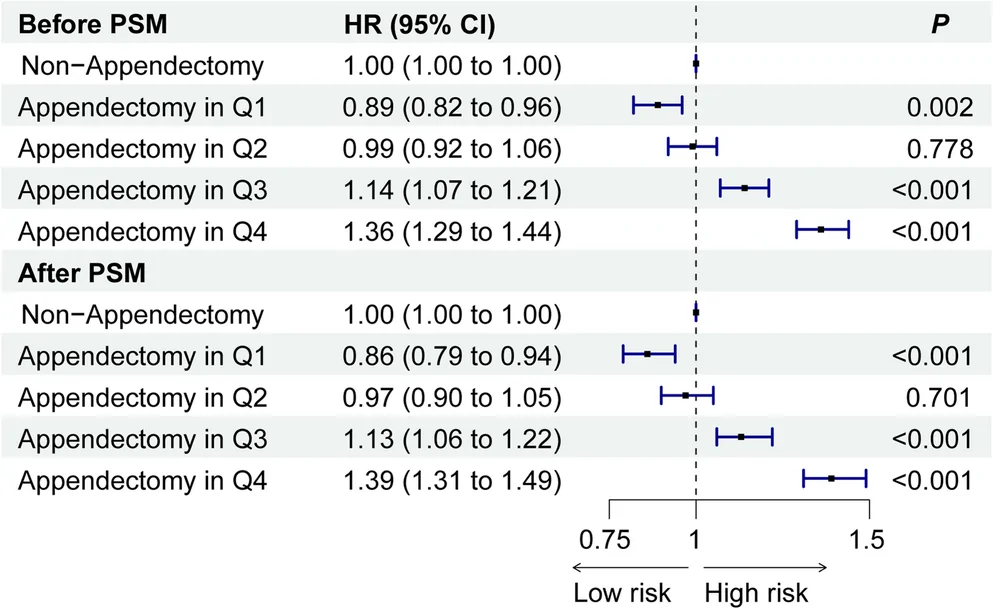
Hazard ratios for pneumonia/influenza associated with appendectomy across quartiles of the weighted modifiable risk-factor score. Abbreviations: Q, quartile; PSM, propensity score matching; HR, hazard ratio; CI, confidence interval

Participants who underwent appendectomy were divided into four groups based on quartiles of the weighted modifiable risk-factor score, and the incidence of pneumonia/influenza per 1000 person-years increased across higher score quartiles. In the fully adjusted model, compared with non-appendectomy participants, appendectomy participants in the first quartile of the weighted modifiable risk-factor score had an 11% (HR: 0.89, 95% CI: 0.82–0.96) lower incidence of pneumonia/influenza, while those in the second quartile showed no significant difference in risk (HR: 0.99, 95% CI: 0.92–1.06). However, participants in the third quartile and the fourth quartile had higher incidence rates of the outcomes, with HRs of 1.14 (95% CI: 1.07–1.21) and 1.36 (95% CI: 1.29–1.44), respectively. Similar trends were observed in the PSM-matched dataset: compared with participants without appendectomy, appendectomy participants in the first quartile of the weighted modifiable risk-factor score had a 14% lower risk of pneumonia/influenza, while appendectomy participants in the highest quartile had a 39% higher risk (Fig. 3 and Supplementary Table 5).

### Sensitivity Analyses and Component-specific Analyses

Consistent results were obtained across multiple sensitivity analyses, including the exclusion of participants with self-reported pneumonia/influenza, those with follow-up periods of less than 2 years, and those with a history of appendicitis. Similarly, multiple imputation for missing covariates and restriction of air pollution exposure levels further supported the robustness of the observed association between appendectomy and the outcomes (Supplementary Tables 6–10, Supplementary Fig. 6). To examine whether the observed association for the composite endpoint was driven by a specific component, we further performed separate analyses using pneumonia and influenza as individual outcomes. The results showed directionally consistent associations for both pneumonia and influenza, suggesting that the association observed for the composite endpoint was not driven solely by either component (Supplementary Table 11, Supplementary Fig. 7).

## Discussion

In this large-scale prospective cohort study based on UK Biobank data, we have three main findings: a). Appendectomy was associated with a higher subsequent risk of pneumonia/influenza. This association remained significant after adjustment for multiple covariates and was also observed in PSM analyses. b). Appendectomy performed before age 10 was not associated with incident pneumonia/influenza, whereas stronger associations were observed for appendectomy performed after age 10, suggesting a potential age-dependent pattern. c). Our findings also indicate that a favorable modifiable risk-factor profile was associated with attenuation of the observed association between appendectomy and pneumonia/influenza. These findings highlight the potential value of long-term respiratory infection prevention strategies among individuals who have undergone appendectomy.

To our knowledge, this study is the first to explore the association between appendectomy and respiratory infections. We found that appendectomy was associated with a higher subsequent risk of pneumonia/influenza, with the appendectomy group having a 10% higher risk of pneumonia/influenza compared with the non-appendectomy group. Although the appendix has historically been considered a vestigial organ, mounting evidence suggests that it plays a role in gut-associated lymphoid tissue and immune homeostasis. Some studies have indicated that the appendix functions to harbor beneficial gut microbiota and aids in modulating immune responses [11, 13, 26, 27]. One biologically plausible explanation is that removal of the appendix may be associated with changes in mucosal immunity and host microbial homeostasis, which could contribute to altered susceptibility to infections, including respiratory infections. For instance, several reports have shown that Clostridium difficile infection and inflammatory bowel disease are more frequently observed after appendectomy [28, 29], highlighting the potential role of the appendix in maintaining gut microbiota balance and the possible consequences of appendectomy for microbial homeostasis. Additionally, immunological studies suggest that the appendix may contribute to the production of immunoglobulins, particularly secretory immunoglobulin A (sIgA) [30]. sIgA plays a crucial role in maintaining mucosal immunity by preventing the adhesion and invasion of pathogens on mucosal surfaces, including the respiratory tract [31, 32]. The removal of the appendix may lead to a reduction in sIgA production, thereby weakening the first line of defense against respiratory pathogens. Similarly, studies on tonsillectomy have shown associations with higher subsequent risks of respiratory infections [33], indicating that lymphoid organs involved in mucosal immunity play an essential role in defending against airborne pathogens. However, few studies have specifically explored the link between appendectomy and pneumonia or influenza. Our findings thus make a valuable contribution to this relatively underexplored field. From a clinical perspective, appendectomy history may help inform long-term risk awareness and individualized preventive care, surgeons should further consider the long-term risks of patients when making appendectomy decisions and conduct a comprehensive assessment to determine the optimal treatment approach.

Our age-stratified analyses revealed heterogeneity in the association between appendectomy and pneumonia/influenza according to age at surgery: appendectomy before 10 years old showed no significant association, whereas surgeries performed after the age of 10 were associated with 10–14% higher risks of pneumonia/influenza, with a potential linear relationship observed in the RCS analysis. This age-dependent divergence may reflect the functional maturation and subsequent involution of gut-associated lymphoid tissue (GALT). During early childhood, both the immune system and gut microbiota are in a dynamic developmental phase [34, 35]. At this stage, the removal of the appendix may be functionally compensated by other lymphoid or gastrointestinal tissues, thereby minimizing its potential association with gut microbiota and long-term immune function. As individuals age, the immune system inevitably undergoes remodeling and senescence [36, 37], resulting in reduced adaptability. Appendectomy performed later in life may therefore be more strongly associated with age-related mucosal immune changes and altered gut microbial composition, which could contribute to greater susceptibility to respiratory infections. Existing research has shown that appendectomy in older adults is associated with reduced gut microbiota diversity and increased disease susceptibility [38, 39]. These findings highlight the importance of considering age at the time of appendectomy when assessing long-term health risks. Long-term risk awareness and preventive strategies, including influenza and pneumococcal vaccination, may be particularly relevant for older adults with a history of appendectomy, because they are inherently more vulnerable to respiratory infections [40, 41]. Future studies are still needed to further investigate the age-specific associations of appendectomy with the gut microbiota and to elucidate the underlying mechanisms.

Given that pneumonia and influenza are strongly influenced by modifiable lifestyle and environmental factors, we further examined whether the association between appendectomy and pneumonia/influenza differed across modifiable risk-factor profiles. Earlier studies have reported that modifiable lifestyle and environmental factors are associated with the incidence and prognosis of multiple diseases [42, 43]. These factors may be related to pneumonia/influenza through various mechanisms. For instance, smoking and air pollutants may damage epithelial cells and impair mucociliary clearance in the respiratory tract, while diet and physical exercise may influence immune function and thereby contribute to pneumonia/influenza susceptibility [44–46]. By calculating a weighted modifiable risk-factor score, we quantified the cumulative burden of modifiable risk factors for each participant. The results showed that appendectomy participants in the lowest weighted modifiable risk-factor score quartile had an 11% lower incidence of pneumonia/influenza than non-appendectomy controls, while those in the highest quartile had a 36% higher incidence. These findings suggest that a favorable modifiable risk-factor profile was associated with attenuation of the observed association between appendectomy and pneumonia/influenza. Overall, these results support the potential value of comprehensive respiratory infection prevention strategies among individuals with a history of appendectomy.

Subgroup analysis revealed significant interactions between appendectomy and sex, as well as between appendectomy and TDI levels. The association with pneumonia/influenza was more pronounced in female participants and individuals with lower TDI. Sex-specific differences may stem from hormonal and immune variations, as estrogen modulates immune responses and may influence post-appendectomy infection risk [47, 48]. Regarding socioeconomic status, this interaction may reflect socioeconomic heterogeneity or residual confounding rather than deprivation-related vulnerability. Therefore, targeted early prevention strategies are essential for high-risk populations, and further research is warranted to validate these associations and inform personalized post-appendectomy care.

A major strength of this study is the use of the UK Biobank, a large prospective cohort with extensive health records and detailed covariate data. This enabled adjustment for a broad range of potential confounders, such as age, sex, smoking status, comorbidities, and socioeconomic variables, thereby enhancing the credibility of our findings. Moreover, our findings have potential clinical implications. First, the observed association between age at appendectomy and subsequent infection risk may aid in identifying vulnerable individuals who may benefit from targeted prevention and monitoring. Second, the role of modifiable risk factors underscores the value of comprehensive respiratory infection prevention strategies following appendectomy. From a public health and policy perspective, our findings suggest that appendectomy history, particularly age at appendectomy, may serve as a simple and accessible marker for identifying individuals at higher subsequent risk of pneumonia/influenza. These results may support the consideration of surgical history in population-level respiratory infection risk assessment, especially for targeted prevention strategies. Public health interventions, including smoking cessation, weight management, promotion of physical activity and healthy diet, reduction of air pollution exposure, and influenza and pneumococcal vaccination, may help reduce the potential excess infection burden among individuals with a history of appendectomy. Importantly, these findings should not be interpreted as discouraging clinically indicated appendectomy, but rather as supporting long-term risk awareness and individualized preventive care after surgery. However, several limitations should be considered. First, we did not account for alterations in risk factors among participants during the follow-up period. Second, information on influenza and pneumococcal vaccination status was unavailable and therefore could not be adjusted for. Given that vaccination may influence the incidence and severity of respiratory infections, unmeasured differences in vaccination uptake may have introduced residual confounding. Third, participants in the UK Biobank may exhibit healthier lifestyle behaviors compared to the general population, leading to a healthy volunteer bias that could result in underestimation of disease risks. Fourth, our study population primarily consists of individuals of European ancestry, which may limit the generalizability of our findings to other ethnic groups. Finally, although we adjusted for a broad range of demographic, socioeconomic, lifestyle, environmental, and clinical covariates and performed multiple sensitivity analyses, residual confounding from unmeasured factors, such as postoperative antibiotic use, healthcare utilization, disease surveillance intensity, or other unmeasured variables, cannot be fully excluded. Future studies should aim to validate these findings in more diverse populations and explore the role of modifiable risk-factor profiles in respiratory infection prevention.

## Conclusion

In conclusion, our study provides epidemiological evidence that appendectomy is associated with a higher subsequent risk of pneumonia/influenza. This association appears to be age-dependent: no significant association was observed when the surgery occurred before age 10, whereas stronger associations were observed for appendectomy performed at older ages. Additionally, a favorable modifiable risk-factor profile was associated with attenuation of the observed association between appendectomy and pneumonia/influenza. From a clinical and public health perspective, these findings may support careful evaluation of appendectomy indications and timing within the context of standard surgical decision-making, while also highlighting the importance of long-term respiratory infection risk awareness and individualized preventive care among individuals with a history of appendectomy.

## Supplementary Information

Below is the link to the electronic supplementary material.

Supplementary Material 1 (PDF 898 KB)

## Data Availability

This study was conducted using the UK Biobank resource under approved project number 473136. UK Biobank makes the source data available to all bona fide researchers for all types of health-related research that is in the public interest, with no individual receiving preferential or exclusive access. For more information on the access procedures, please visit the UK Biobank website.

## Abbreviations

BMI: Body mass index
HR: Hazard ratio
CI: Confidence interval
PSM: Propensity score matching
TDI: Townsend Deprivation Index
COPD: Chronic obstructive pulmonary disease
CHD: Coronary heart disease
RCS: Restricted cubic spline
