# Supplementary material for: Age at Appendectomy, Pneumonia/Influenza Risk, and The Role of Modifiable Risk-factor Profiles
Source: J Epidemiol Glob Health. 2026 Jul 11;16(1):132. doi: 10.1007/s44197-026-00612-0 (PMC13650216; doi:10.1007/s44197-026-00612-0)
Supplement: Supplementary file 1 [file 44197_2026_612_MOESM1_ESM.pdf]

Supplementary Table 1. The β coefficients of each variable.

Variables	β coefficient
$PM_{2.5}$	0.090
PM_{10}	0.032
PM_{coarse}	0.025
NO_x	0.005
NO_2	0.008
Smoking status	0.640
Alcohol consumption	0.059
BMI: 25-30 kg/m^2	0.208
BMI: >30 kg/m^2	0.586
Diet	0.132
Physical activity	0.222

The β coefficients were obtained by including each variable individually in regression analyses with outcomes. The "<25 kg/m^2" group in BMI was used as the reference group. Abbreviations: BMI, body mass index.

Supplementary Table 2. Definitions of covariates.

Variables	Definition
Age	Continuous variable. Data-field: 34, 52, 53
Sex	Male and female. Data-field: 31
Ethnicity	White and non-white. Data-field: 21000
Education level	Low: CSEs or equivalent, O levels/GCSEs or equivalent; Medium: A levels/AS levels or equivalent, NVQ or HND or HNC or equivalent; High: College or University degree, other professional qualifications eg: nursing, teaching; Unknown/Missing. Data-field: 6138
Body mass index	Weight in kilograms (kg) divided by the square of the height in meters (m^2). Data-field: 21001
Townsend deprivation index	This indicator reflected the integrated condition of housing, employment, and social class individually. Data-field: 22189
Smoking status	Current smoker, past smoker and never smoker. Data-field: 20116
Alcohol consumption	≤4 times a week and daily or almost daily. Data-field: 1558
Physical activity	Regular physical activity was defined as engagement in at least 150 minutes of walking, moderate activity per week, or 75 minutes of vigorous activity. Data-field: 864, 874, 884, 894, 904, 914, 22036
Diet	Total fruit and vegetable intake: > 4.5 pieces or servings a week. Total fish intake: > 2 per week. Processed and red meat intake: 2 or fewer times intake of processed meat per week and 5 or fewer times intake of red meat per week. Healthy diet defined as ≥ 2 of the healthy food items. Data-field: 1289, 1299, 1309, 1319, 1329, 1339, 1349, 1369, 1379, 1389
$PM_{2.5}$	Fine particulate matter with diameter < 2.5μm. Data-field: 24006
PM_{10}	Particulate matter with diameter < 10μm. Data-field: 24005, 24019
PM_{coarse}	Coarse particulate matter with diameter between 2.5μm and 10μm. Data-field: 24008
NO_2	Nitrogen dioxide. Data-field: 24003, 24016, 24017, 24018
NO_x	Nitrogen Oxides. Data-field: 24004
Hypertension	Based on the 10th International Classification of Diseases codes. Data-field: 41270 (I10-I13, I15), 131286, 131288, 131290, 131292, 131294
Diabetes	Based on the 10th International Classification of Diseases codes. Data-field: 41270 (E10-E14), 130706, 130708, 130710, 130712, 130714

COPD	Based on the Algorithmically-Defined Outcomes (ADOs). Data-field: 42016
Asthma	Based on the Algorithmically-Defined Outcomes (ADOs). Data-field: 42014
CHD	Based on the 10th International Classification of Diseases codes. Data-field: 41270 (I20-I25), 131296, 131298, 131300, 131302, 131304, 131306
Stroke	Based on the Algorithmically-Defined Outcomes (ADOs). Data-field: 42006
Appendectomy	Operative procedures-3, operative procedures-4, operation code. Data-field: 41273, 41272, 20004
Pneumonia/Influenza	Based on the 10th International Classification of Diseases codes. Data-field: 41270 (J09-J18), 131438, 131440, 131442, 131444, 131446, 131448,131450, 131452, 131554, 131456

Abbreviations: ICD: international classification of diseases. Data Field: a data-field is the fundamental block of data held within the UK Biobank repository and identifies the results of a single question, measurement or result (or part thereof); COPD, chronic obstructive pulmonary disease; CHD, coronary heart disease.

Supplementary Table 3. Baseline characteristics of included participants grouped by age at appendectomy.

Characteristics	Non-Appendectomy (N=357636)	Appendectomy (N=46941)				*P*
		Age at surgery < 10 years (N=7142)	Age at surgery 10-20 years (N=21449)	Age at surgery 20-30 years (N=9611)	Age at surgery ≥ 30 years (N=8739)	
**Age, mean (SD)**	56.8 (8.1)	58.0 (7.5)	58.3 (7.7)	58.9 (7.7)	59.1 (7.5)	<0.001
**Male, n (%)**	164382 (46.0)	3419 (47.9)	9123 (42.5)	3705 (38.5)	3724 (42.6)	<0.001
**Ethnicity, White, n (%)**	338885 (94.8)	7023 (98.3)	20838 (97.2)	9201 (95.7)	8380 (95.9)	<0.001
**Education Level, n (%)**						<0.001
High	172086 (48.1)	3984 (55.8)	10687 (49.8)	4777 (49.7)	4098 (46.9)	
Medium	66956 (18.7)	1172 (16.4)	3843 (17.9)	1627 (16.9)	1547 (17.7)	
Low	62805 (17.6)	1122 (15.7)	3583 (16.7)	1656 (17.2)	1480 (16.9)	
None of the above	55789 (15.6)	864 (12.1)	3336 (15.6)	1551 (16.1)	1614 (18.5)	
**BMI category, n (%)**						<0.001
<25 kg/m^2	120783 (33.8)	2445 (34.2)	6888 (32.1)	2985 (31.1)	2669 (30.5)	
25-30 kg/m^2	152685 (42.7)	3040 (42.6)	9085 (42.4)	4089 (42.5)	3789 (43.4)	

>30 kg/m^2	84168 (23.5)	1657 (23.2)	5476 (25.5)	2537 (26.4)	2281 (26.1)	
**Townsend deprivation index, mean (SD)**	-1.4 (3.0)	-1.5 (3.0)	-1.5 (3.0)	-1.5 (2.9)	-1.6 (2.9)	<0.001
**Smoking status, n (%)**						<0.001
Never or Past	322297 (90.1)	6412 (89.8)	19126 (89.2)	8660 (90.1)	7873 (90.1)	
Current	35339 (9.9)	730 (10.2)	2323 (10.8)	951 (9.9)	866 (9.9)	
**Alcohol consumption, n (%)**						<0.001
≤4 times a week	282977 (79.1)	5424 (75.9)	16731 (78.0)	7581 (78.9)	6883 (78.8)	
Daily or almost daily	74659 (20.9)	1718 (24.1)	4718 (22.0)	2030 (21.1)	1856 (21.2)	
**Physical activity, n (%)**	277562 (77.6)	5497 (77.0)	16500 (76.9)	7445 (77.5)	6702 (76.7)	0.031
**Healthful diet, n (%)**	200066 (55.9)	4059 (56.8)	12501 (58.3)	5873 (61.1)	5146 (58.9)	<0.001
**$PM_{2.5}$, mean (SD)**	10.0 (1.1)	9.9 (1.1)	10.0 (1.1)	10.0 (1.1)	9.9 (1.0)	<0.001
**PM_{10}, mean (SD)**	19.3 (1.9)	19.3 (2.0)	19.3 (2.0)	19.3 (2.0)	19.2 (1.9)	<0.001
**PM_{coarse}, mean (SD)**	6.4 (0.9)	6.4 (0.9)	6.4 (0.9)	6.4 (0.9)	6.4 (0.9)	0.080
**NO_2, mean (SD)**	29.1 (9.2)	29.0 (9.7)	28.9 (9.3)	28.8 (9.2)	28.4 (8.9)	<0.001

**NO_x, mean (SD)**	43.8 (15.5)	43.0 (15.7)	43.4 (15.7)	43.5 (15.8)	42.9 (15.4)	<0.001
**Air pollution score, mean (SD)**	13.4 (1.8)	13.4 (1.9)	13.4 (1.9)	13.4 (1.9)	13.3 (1.8)	<0.001
**Prevalent disease, n (%)**						
Hypertension	92831 (26.0)	1870 (26.2)	6118 (28.5)	2885 (30.0)	2660 (30.4)	<0.001
Diabetes	17332 (4.8)	339 (4.7)	1221 (5.7)	590 (6.1)	516 (5.9)	<0.001
COPD	5065 (1.4)	114 (1.6)	441 (2.1)	194 (2.0)	176 (2.0)	<0.001
Asthma	39190 (11.0)	775 (10.9)	2668 (12.4)	1212 (12.6)	1056 (12.1)	<0.001
CHD	17273 (4.8)	395 (5.5)	1386 (6.5)	621 (6.5)	601 (6.9)	<0.001
Stroke	4702 (1.3)	101 (1.4)	364 (1.7)	180 (1.9)	140 (1.6)	<0.001

Data were expressed as mean (SD) and count (%). *P* values were computed using one-way analysis of variance or chi-square tests, as appropriate. Abbreviations: SD, standard deviation; BMI, body mass index; COPD, chronic obstructive pulmonary disease; CHD, coronary heart disease.

Supplementary Table 4. Baseline characteristics of included participants grouped by weighted modifiable risk-factor score.

Characteristics	Non-Appendectomy (N=357636)	Appendectomy (N=46941)				*P*
		Weighted modifiable risk-factor score Q1 (N=11691)	Weighted modifiable risk-factor score Q2 (N=11518)	Weighted modifiable risk-factor score Q3 (N=11855)	Weighted modifiable risk-factor score Q4 (N=11877)	
**Age, mean (SD)**	56.8 (8.1)	59.2 (7.4)	59.0 (7.7)	58.5 (7.7)	57.5 (7.8)	<0.001
**Male, n (%)**	164382 (46.0)	4166 (35.6)	4913 (42.7)	5275 (44.5)	5617 (47.3)	<0.001
**Ethnicity, White, n (%)**	338885 (94.8)	11493 (98.3)	11245 (97.6)	11460 (96.7)	11244 (94.7)	<0.001
**Education Level, n (%)**						<0.001
High	172086 (48.1)	6626 (56.7)	5854 (50.8)	5813 (49.0)	5253 (44.2)	
Medium	66956 (18.7)	1816 (15.5)	2016 (17.5)	2130 (18.0)	2227 (18.8)	
Low	62805 (17.6)	1917 (16.4)	1942 (16.9)	1962 (16.5)	2020 (17.0)	
None of the above	55789 (15.6)	1332 (11.4)	1706 (14.8)	1950 (16.4)	2377 (20.0)	
**BMI category, n (%)**						<0.001
<25 kg/m^2	120783 (33.8)	7172 (61.3)	3820 (33.2)	2278 (19.2)	1717 (14.5)	

25-30 kg/m^2	152685 (42.7)	4429 (37.9)	6600 (57.3)	5437 (45.9)	3537 (29.8)	
>30 kg/m^2	84168 (23.5)	90 (0.8)	1098 (9.5)	4140 (34.9)	6623 (55.8)	
**Townsend deprivation index, mean (SD)**	-1.4 (3.0)	-2.8 (2.1)	-2.1 (2.5)	-1.3 (2.8)	0.2 (3.4)	<0.001
**Smoking status, n (%)**						<0.001
Never or Past	322297 (90.1)	11691 (100.0)	11407 (99.0)	11155 (94.1)	7818 (65.8)	
Current	35339 (9.9)	0 (0.0)	111 (1.0)	700 (5.9)	4059 (34.2)	
**Alcohol consumption, n (%)**						0.003
≤4 times a week	282977 (79.1)	9130 (78.1)	9112 (79.1)	9217 (77.7)	9160 (77.1)	
Daily or almost daily	74659 (20.9)	2561 (21.9)	2406 (20.9)	2638 (22.3)	2717 (22.9)	
**Physical activity, n (%)**	277562 (77.6)	10702 (91.5)	9511 (82.6)	8643 (72.9)	7288 (61.4)	<0.001
**Healthful diet, n (%)**	200066 (55.9)	8730 (74.7)	7078 (61.5)	6544 (55.2)	5227 (44.0)	<0.001
**$PM_{2.5}$, mean (SD)**	10.0 (1.1)	9.1 (0.7)	9.8 (0.7)	10.1 (0.9)	10.8 (1.1)	<0.001
**PM_{10}, mean (SD)**	19.3 (1.9)	17.7 (1.4)	19.0 (1.5)	19.6 (1.7)	20.6 (2.0)	<0.001
**PM_{coarse}, mean (SD)**	6.4 (0.9)	6.2 (0.8)	6.3 (0.8)	6.5 (0.9)	6.7 (1.0)	<0.001

**NO_2, mean (SD)**	29.1 (9.2)	21.6 (5.2)	27.3 (6.3)	30.4 (7.9)	35.8 (10.5)	<0.001
**NO_x, mean (SD)**	43.8 (15.5)	31.6 (7.8)	40.5 (9.2)	45.4 (12.0)	55.4 (19.7)	<0.001
**Air pollution score, mean (SD)**	13.4 (1.8)	11.8 (1.0)	13.1 (1.1)	13.7 (1.4)	14.9 (2.0)	<0.001
**Prevalent disease, n (%)**						
Hypertension	92831 (26.0)	2446 (20.9)	3033 (26.3)	3767 (31.8)	4287 (36.1)	<0.001
Diabetes	17332 (4.8)	260 (2.2)	476 (4.1)	779 (6.6)	1151 (9.7)	<0.001
COPD	5065 (1.4)	108 (0.9)	161 (1.4)	259 (2.2)	397 (3.3)	<0.001
Asthma	39190 (11.0)	1256 (10.7)	1290 (11.2)	1530 (12.9)	1635 (13.8)	<0.001
CHD	17273 (4.8)	470 (4.0)	647 (5.6)	851 (7.2)	1035 (8.7)	<0.001
Stroke	4702 (1.3)	125 (1.1)	167 (1.4)	217 (1.8)	276 (2.3)	<0.001

Data were expressed as mean (SD) and count (%). *P* values were computed using one-way analysis of variance or chi-square tests, as appropriate. Abbreviations: Q, quartiles; SD, standard deviation; BMI, body mass index; COPD, chronic obstructive pulmonary disease; CHD, coronary heart disease.

Supplementary Table 5. Hazard ratios for pneumonia/influenza associated with appendectomy across quartiles of the weighted modifiable risk-factor score.

Exposure	Before PSM				After PSM			
	Event/Total	Incidence (per 1000 person-year)	Fully adjusted Model HR (95% CI)	*P*	Event/Total	Incidence (per 1000 person-year)	Fully adjusted Model HR (95% CI)	*P*
**Non-Appendectomy**	23786/357636	5.1	1.00		3468/46939	5.7	1.00	
**Appendectomy with score in Q1**	647/11691	3.7	0.89 (0.82-0.96)	0.002	647/11691	4.0	0.86 (0.79-0.94)	<0.001
**Appendectomy with score in Q2**	796/11518	4.7	0.99 (0.92-1.06)	0.778	796/11518	5.1	0.97 (0.90-1.05)	0.701
**Appendectomy with score in Q3**	1019/11855	6.1	1.14 (1.07-1.21)	<0.001	1019/11855	6.7	1.13 (1.06-1.22)	<0.001
**Appendectomy with score in Q4**	1341/11877	8.7	1.36 (1.29-1.44)	<0.001	1340/11875	9.4	1.39 (1.31-1.49)	<0.001

Fully adjusted Model was adjusted for age, sex, ethnicity, Townsend deprivation index, education level, prevalent diseases (hypertension, diabetes, COPD, asthma, CHD, stroke). Abbreviations: Q, quartiles; PSM, propensity score matching; HR, hazard ratio; CI, confidence interval; COPD, chronic obstructive pulmonary disease; CHD, coronary heart disease. In this table, "score" refers to the weighted modifiable risk-factor score.

Supplementary Table 6. Sensitivity analysis after excluding self-reported pneumonia/influenza participants.

Exposure	Event/Total	Incidence (per 1000 person-year)	Fully adjusted Model HR (95% CI)	*P*
**Appendectomy and risk of pneumonia/influenza**				
Non-Appendectomy	23539/357389	5.1	1.00	
Appendectomy	3758/46896	5.7	1.10 (1.06-1.14)	<0.001
**Age at appendectomy and risk of pneumonia/influenza**				
Non-Appendectomy	23539/357389	5.1	1.00	
Age at surgery < 10 years	492/7138	5.0	1.01 (0.92-1.10)	0.844
Age at surgery 10-20 years	1702/21427	5.7	1.09 (1.04-1.15)	<0.001
Age at surgery 20-30 years	817/9599	5.9	1.13 (1.05-1.21)	<0.001
Age at surgery ≥ 30 years	747/8732	6.0	1.13 (1.06-1.22)	<0.001
			$P_{trend} < 0.001$	
**Weighted modifiable risk-factor score and risk of pneumonia/influenza**				
Non-Appendectomy	23539/357389	5.1	1.00	
Appendectomy with score in Q1	625/11663	3.5	0.87 (0.80-0.94)	<0.001
Appendectomy with score in Q2	789/11526	4.6	0.99 (0.92-1.06)	0.748
Appendectomy with score in Q3	1012/11841	6.1	1.14 (1.07-1.22)	<0.001
Appendectomy with score in Q4	1332/11866	8.6	1.36 (1.29-1.44)	<0.001

Fully adjusted Model was adjusted for age, sex, ethnicity, Townsend deprivation index, education level, BMI, smoking status, alcohol consumption, physical activity, healthful diet, air pollution score, prevalent diseases (hypertension, diabetes, COPD, asthma, CHD, stroke). In analyses stratified by weighted modifiable risk-factor score quartiles, covariates already incorporated into the score were excluded from the fully adjusted model. Abbreviations: Q, quartiles; HR, hazard ratio; CI, confidence interval; BMI, body mass index; COPD, chronic obstructive pulmonary disease; CHD, coronary heart disease. P_{trend} was performed by treating the variables continuously.

Supplementary Table 7. Sensitivity analysis after excluding incident cases during the first two years of follow-up.

Exposure	Event/Total	Incidence (per 1000 person-year)	Fully adjusted Model HR (95% CI)	*P*
**Appendectomy and risk of pneumonia/influenza**				
Non-Appendectomy	21576/354057	4.7	1.00	
Appendectomy	3418/46368	5.1	1.08 (1.05-1.12)	<0.001
**Age at appendectomy and risk of pneumonia/influenza**				
**Non-Appendectomy**	21576/354057	4.7	1.00	
Age at surgery < 10 years	445/7061	4.5	0.99 (0.90-1.09)	0.874
Age at surgery 10-20 years	1536/21183	5.1	1.07 (1.02-1.13)	0.008
Age at surgery 20-30 years	748/9495	5.3	1.12 (1.04-1.21)	0.002
Age at surgery ⩾ 30 years	689/8629	5.5	1.14 (1.05-1.23)	<0.001
			$P_{trend} < 0.001$	
**Weighted modifiable risk-factor score and risk of pneumonia/influenza**				
Non-Appendectomy	21576/354057	4.7	1.00	
Appendectomy with score in Q1	556/11527	3.1	0.84 (0.77-0.91)	<0.001
Appendectomy with score in Q2	721/11413	4.2	0.98 (0.91-1.06)	0.594
Appendectomy with score in Q3	930/11661	5.6	1.14 (1.07-1.22)	<0.001
Appendectomy with score in Q4	1211/11767	7.8	1.35 (1.27-1.43)	<0.001

Fully adjusted Model was adjusted for age, sex, ethnicity, Townsend deprivation index, education level, BMI, smoking status, alcohol consumption, physical activity, healthful diet, air pollution score, prevalent diseases (hypertension, diabetes, COPD, asthma, CHD, stroke). In analyses stratified by weighted modifiable risk-factor score quartiles, covariates already incorporated into the score were excluded from the fully adjusted model. Abbreviations: Q, quartiles; HR, hazard ratio; CI, confidence interval; BMI, body mass index; COPD, chronic obstructive pulmonary disease; CHD, coronary heart disease. P_{trend} was performed by treating the variables continuously.

Supplementary Table 8. Sensitivity analysis after excluding patients with a history of appendicitis at baseline.

Exposure	Event/Total	Incidence (per 1000 person-year)	Fully adjusted Model HR (95% CI)	*P*
**Appendectomy and risk of pneumonia/influenza**				
Non-Appendectomy	23338/350830	5.1	1.00	
Appendectomy	3734/46066	5.7	1.10 (1.06-1.14)	<0.001
**Age at appendectomy and risk of pneumonia/influenza**				
Non-Appendectomy	23338/350830	5.1	1.00	
Age at surgery < 10 years	487/6997	5.1	1.01 (0.92-1.10)	0.851
Age at surgery 10-20 years	1696/21053	5.8	1.10 (1.05-1.15)	<0.001
Age at surgery 20-30 years	811/9439	5.9	1.13 (1.05-1.21)	<0.001
Age at surgery ≥ 30 years	740/8577	6.0	1.14 (1.06-1.22)	<0.001
			$P_{trend} < 0.001$	
**Weighted modifiable risk-factor score and risk of pneumonia/influenza**				
Non-Appendectomy	23338/350830	5.1	1.00	
Appendectomy with score in Q1	634/11463	3.7	0.88 (0.82-0.96)	0.002
Appendectomy with score in Q2	783/11317	4.7	0.99 (0.92-1.06)	0.797
Appendectomy with score in Q3	994/11633	6.1	1.13 (1.06-1.21)	<0.001
Appendectomy with score in Q4	1323/11653	8.7	1.37 (1.30-1.45)	<0.001

Fully adjusted Model was adjusted for age, sex, ethnicity, Townsend deprivation index, education level, BMI, smoking status, alcohol consumption, physical activity, healthful diet, air pollution score, prevalent diseases (hypertension, diabetes, COPD, asthma, CHD, stroke). In analyses stratified by weighted modifiable risk-factor score quartiles, covariates already incorporated into the score were excluded from the fully adjusted model. Abbreviations: Q, quartiles; HR, hazard ratio; CI, confidence interval; BMI, body mass index; COPD, chronic obstructive pulmonary disease; CHD, coronary heart disease. P_{trend} was performed by treating the variables continuously.

Supplementary Table 9. Sensitivity analysis after performing multiple imputation.

Exposure	Event/Total	Incidence (per 1000 person-year)	Fully adjusted Model HR (95% CI)	*P*
**Appendectomy and risk of pneumonia/influenza**				
Non-Appendectomy	29488/422031	5.4	1.00	
Appendectomy	4709/55563	6.0	1.10 (1.06-1.13)	<0.001
**Age at appendectomy and risk of pneumonia/influenza**				
Non-Appendectomy	29488/422031	5.4	1.00	
Age at surgery < 10 years	607/8426	5.3	1.01 (0.93-1.09)	0.852
Age at surgery 10-20 years	2146/25361	6.0	1.09 (1.04-1.14)	<0.001
Age at surgery 20-30 years	1021/11484	6.2	1.12 (1.05-1.19)	<0.001
Age at surgery ≥ 30 years	935/10292	6.4	1.14 (1.07-1.22)	<0.001
			$P_{\text{trend}} < 0.001$	
**Weighted modifiable risk-factor score and risk of pneumonia/influenza**				
Non-Appendectomy	29488/422031	5.4	1.00	
Appendectomy with score in Q1	844/14091	4.0	0.89 (0.84-0.96)	0.001
Appendectomy with score in Q2	1016/13731	5.1	1.02 (0.96-1.08)	0.609
Appendectomy with score in Q3	1151/13689	6.0	1.07 (1.01-1.13)	0.027
Appendectomy with score in Q4	1698/14052	9.3	1.39 (1.32-1.46)	<0.001

Fully adjusted Model was adjusted for age, sex, ethnicity, Townsend deprivation index, education level, BMI, smoking status, alcohol consumption, physical activity, healthful diet, air pollution score, prevalent diseases (hypertension, diabetes, COPD, asthma, CHD, stroke). In analyses stratified by weighted modifiable risk-factor score quartiles, covariates already incorporated into the score were excluded from the fully adjusted model. Abbreviations: Q, quartiles; HR, hazard ratio; CI, confidence interval; BMI, body mass index; COPD, chronic obstructive pulmonary disease; CHD, coronary heart disease. P_{trend} was performed by treating the variables continuously.

Supplementary Table 10. Sensitivity analysis after limiting air pollution levels.

Exposure	Event/Total	Incidence (per 1000 person-year)	Fully adjusted Model HR (95% CI)	*P*
**Appendectomy and risk of pneumonia/influenza**				
Non-Appendectomy	20959/318160	5.1	1.00	
Appendectomy	3394/41941	5.7	1.11 (1.07-1.15)	<0.001
**Age at appendectomy and risk of pneumonia/influenza**				
Non-Appendectomy	20959/318160	5.1	1.00	
Age at surgery < 10 years	442/6286	5.1	1.03 (0.94-1.13)	0.573
Age at surgery 10-20 years	1535/19156	5.7	1.10 (1.05-1.16)	<0.001
Age at surgery 20-30 years	736/8595	5.9	1.15 (1.07-1.23)	<0.001
Age at surgery ≥ 30 years	681/7904	6.0	1.14 (1.06-1.23)	<0.001
			$P_{trend} < 0.001$	
**Weighted modifiable risk-factor score and risk of pneumonia/influenza**				
Non-Appendectomy	20959/318160	5.1	1.00	
Appendectomy with score in Q1	575/10486	3.7	0.88 (0.81-0.96)	0.002
Appendectomy with score in Q2	707/10267	4.7	0.99 (0.92-1.07)	0.792
Appendectomy with score in Q3	885/10390	6.1	1.14 (1.06-1.22)	<0.001
Appendectomy with score in Q4	1227/10798	8.7	1.40 (1.32-1.48)	<0.001

Fully adjusted Model was adjusted for age, sex, ethnicity, Townsend deprivation index, education level, BMI, smoking status, alcohol consumption, physical activity, healthful diet, air pollution score, prevalent diseases (hypertension, diabetes, COPD, asthma, CHD, stroke). In analyses stratified by weighted modifiable risk-factor score quartiles, covariates already incorporated into the score were excluded from the fully adjusted model. Abbreviations: Q, quartiles; HR, hazard ratio; CI, confidence interval; BMI, body mass index; COPD, chronic obstructive pulmonary disease; CHD, coronary heart disease. P_{trend} was performed by treating the variables continuously.

Supplementary Table 11. Component-specific analyses of appendectomy and age at appendectomy for pneumonia and influenza.

Exposure	Event/Total	Incidence (per 1000 person-year)	Fully adjusted Model HR (95% CI)	*P*
**Appendectomy and risk of pneumonia**				
Non-Appendectomy	21821/363372	4.6	1.00	
Appendectomy	3489/47773	5.1	1.08 (1.05-1.12)	<0.001
**Age at appendectomy and risk of pneumonia**				
Non-Appendectomy	21821/363372	4.6	1.00	
Age at surgery < 10 years	458/7256	4.6	1.01 (0.92-1.10)	0.879
Age at surgery 10-20 years	1588/21830	5.1	1.09 (1.03-1.14)	0.001
Age at surgery 20-30 years	758/9792	5.2	1.11 (1.03-1.19)	0.005
Age at surgery ≥ 30 years	685/8895	5.3	1.10 (1.02-1.19)	0.011
			$P_{trend} < 0.001$	
**Appendectomy and risk of influenza**				
Non-Appendectomy	3122/366088	0.6	1.00	
Appendectomy	519/48470	0.8	1.22 (1.11-1.34)	<0.001
**Age at appendectomy and risk of influenza**				
Non-Appendectomy	3122/366088	0.6	1.00	
Age at surgery < 10 years	66/7395	0.7	1.08 (0.85-1.38)	0.530
Age at surgery 10-20 years	229/22162	0.8	1.16 (1.02-1.33)	0.026
Age at surgery 20-30 years	121/9905	0.9	1.37 (1.14-1.64)	<0.001
Age at surgery ≥ 30 years	103/9008	0.9	1.29 (1.06-1.57)	0.012
			$P_{trend} < 0.001$	

Fully adjusted Model was adjusted for age, sex, ethnicity, Townsend deprivation index, education level, BMI, smoking status, alcohol consumption, physical activity, healthful diet, air pollution score, prevalent diseases (hypertension,

diabetes, COPD, asthma, CHD, stroke). Abbreviations: HR, hazard ratio; CI, confidence interval; BMI, body mass index; COPD, chronic obstructive pulmonary disease; CHD, coronary heart disease. P_{trend} was performed by treating the variables continuously.

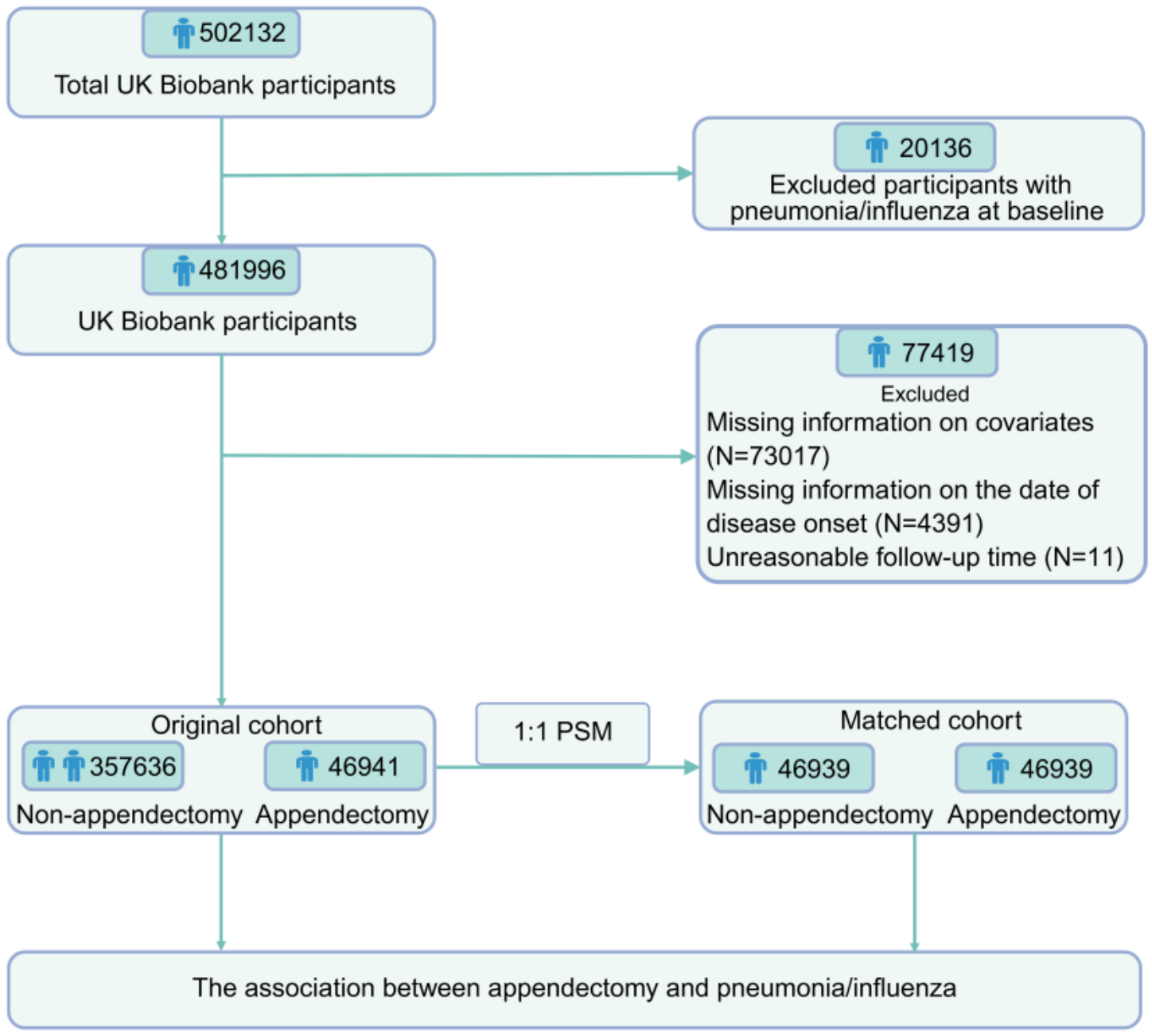

Supplementary Figure 1. Flowchart for included participants. Abbreviations: PSM, propensity score matching.

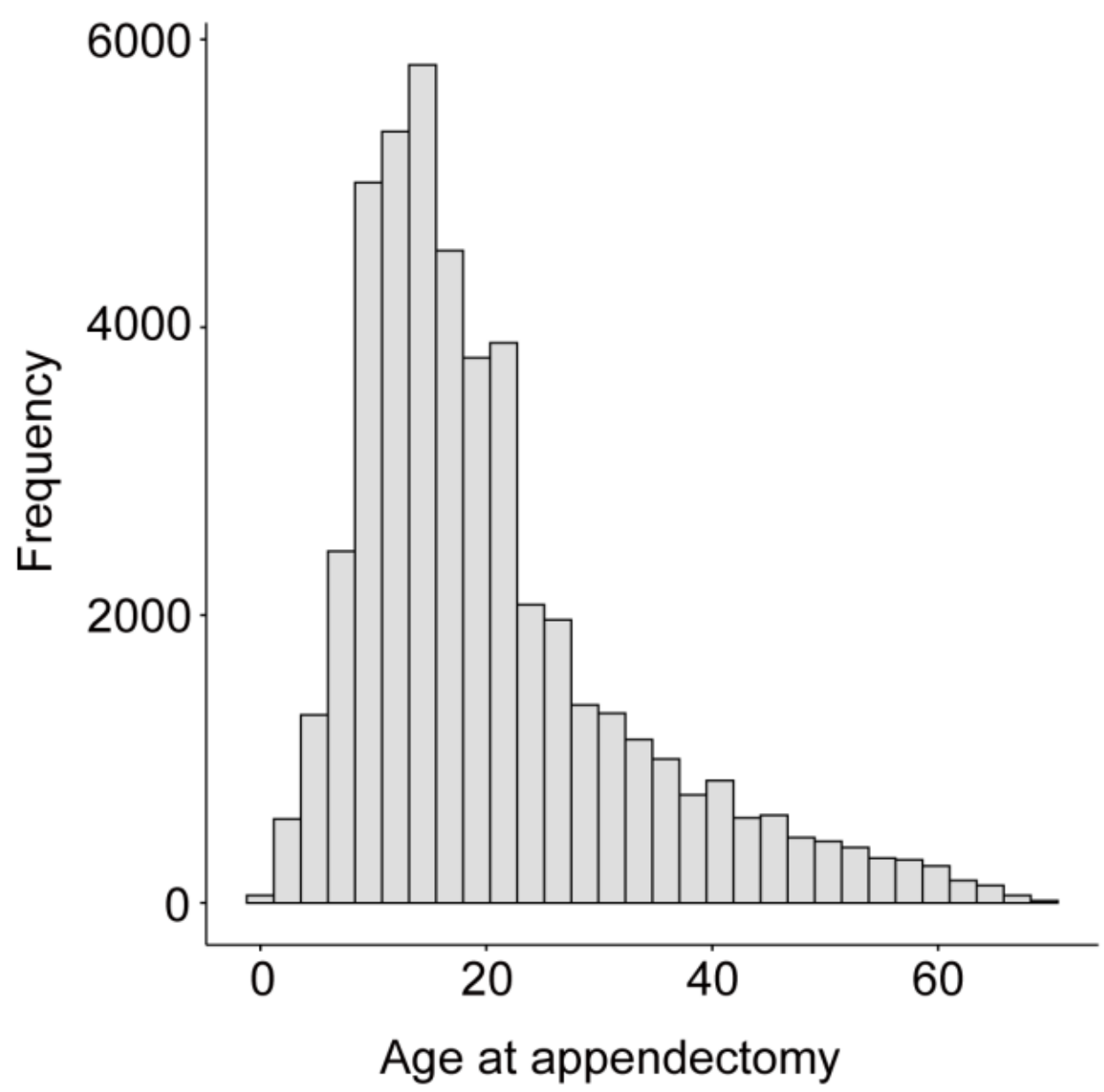

Supplementary Figure 2. Age at surgery among participants who underwent appendectomy.

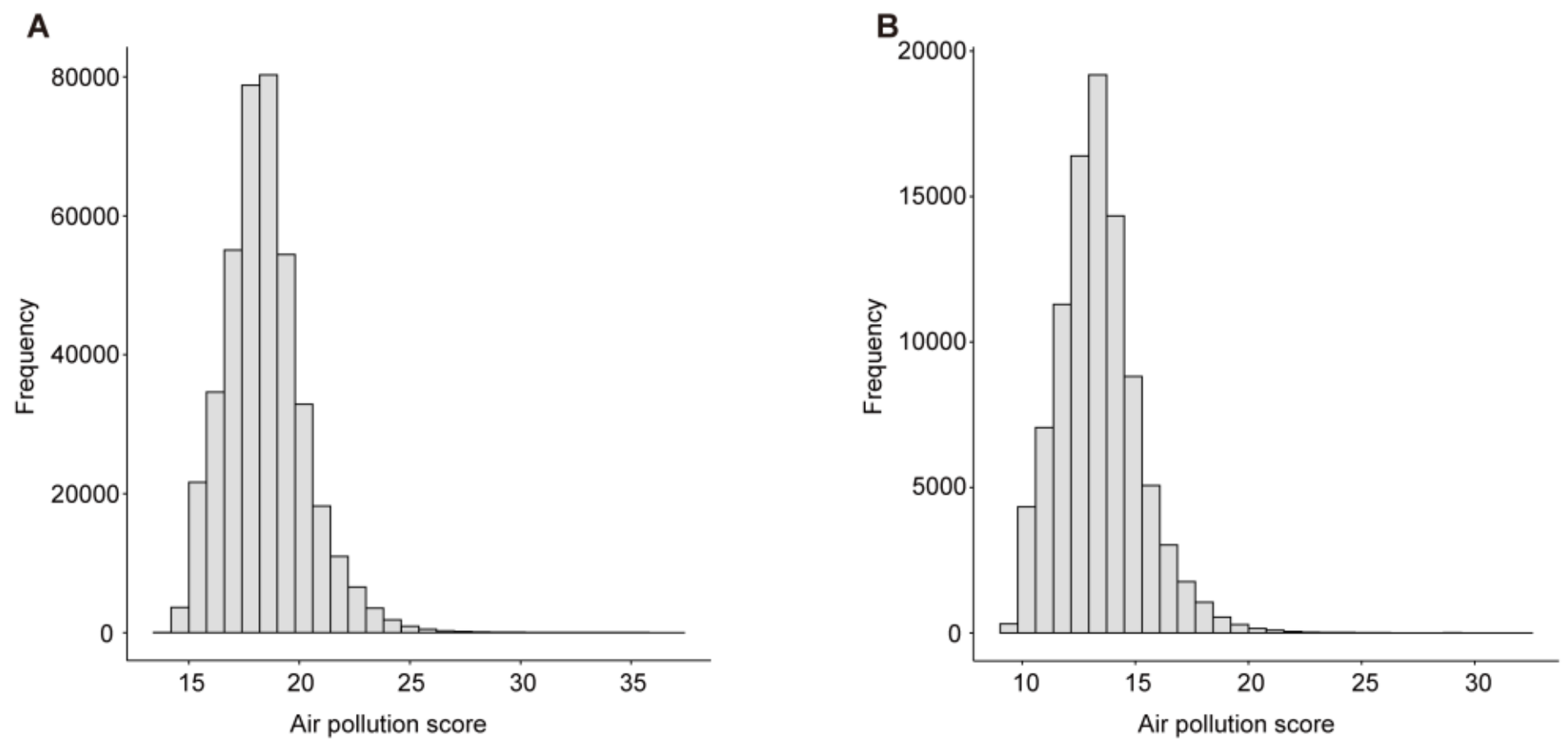

Supplementary Figure 3. Distribution of air pollution score among participants. A. Before PSM; B. After PSM. Abbreviations: PSM, propensity score matching.

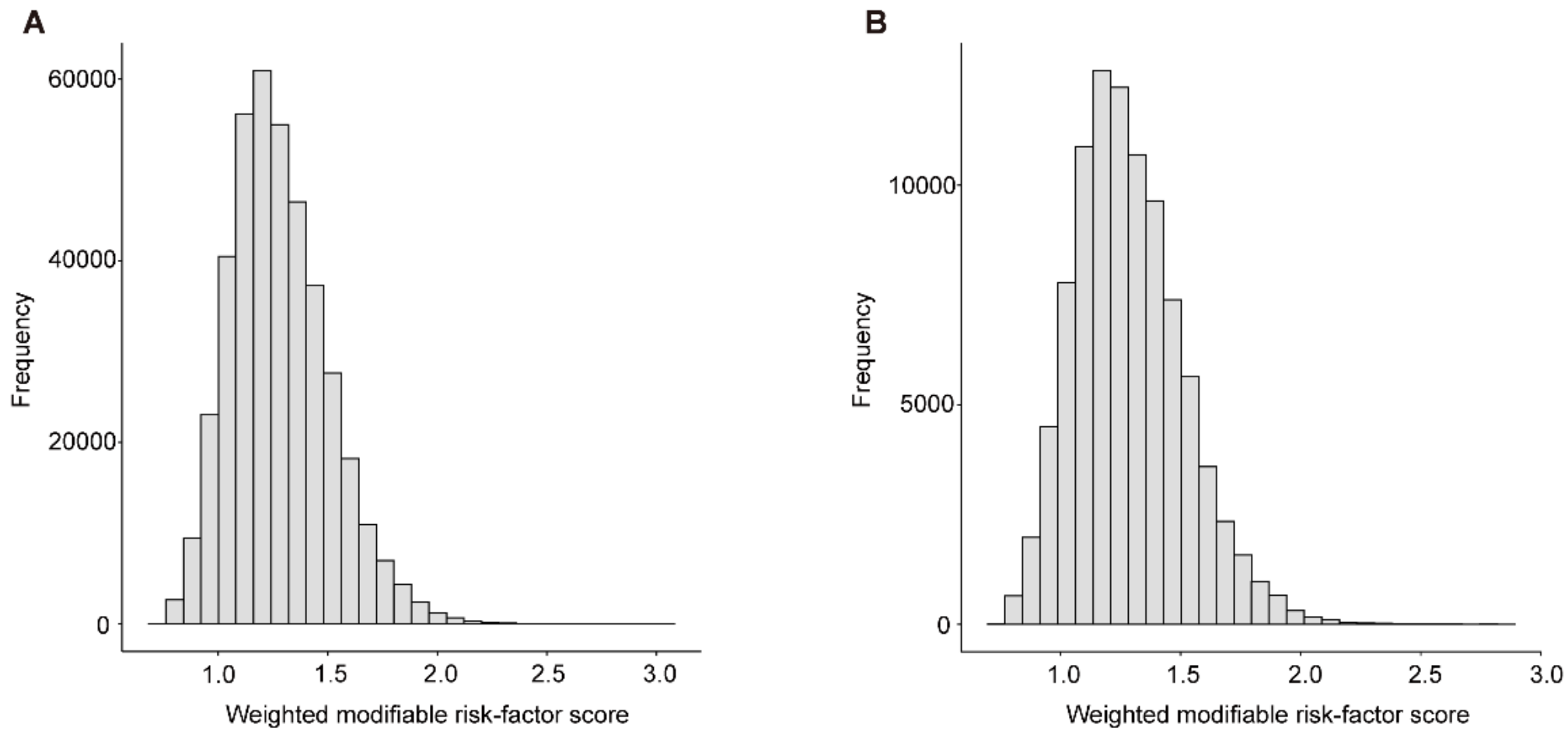

Supplementary Figure 4. Distribution of the weighted modifiable risk-factor score among participants. A. Before PSM; B. After PSM. Abbreviations: PSM, propensity score matching.

Subgroup	Event/Total	HR (95% CI)	*Pi*
Sex			0.033
Female	12295/220224	1.14 (1.09 to 1.20)	
Male	15294/184353	1.06 (1.01 to 1.11)	
Age			0.060
<60	10151/229863	1.16 (1.09 to 1.23)	
≥60	17438/174714	1.11 (1.06 to 1.15)	
BMI			0.333
<25	7285/135770	1.13 (1.05 to 1.21)	
25−30	11316/172688	1.08 (1.03 to 1.14)	
≥30	8988/96119	1.09 (1.03 to 1.16)	
Smoking status			0.733
Never or past	23002/364368	1.10 (1.06 to 1.14)	
Current	4587/40209	1.10 (1.01 to 1.19)	
Alcohol consumption			0.509
≤4 times a week	21544/319596	1.11 (1.06 to 1.15)	
Daily or almost daily	6045/84981	1.07 (1.00 to 1.15)	
TDI			0.049
<-1.438	14704/244117	1.13 (1.08 to 1.18)	
≥-1.438	12885/160460	1.07 (1.01 to 1.12)	
COPD			0.807
No	25968/398587	1.10 (1.06 to 1.14)	
Yes	1621/5990	1.08 (0.95 to 1.23)	
Asthma			0.494
No	23184/359676	1.09 (1.05 to 1.13)	
Yes	4405/44901	1.13 (1.04 to 1.23)	

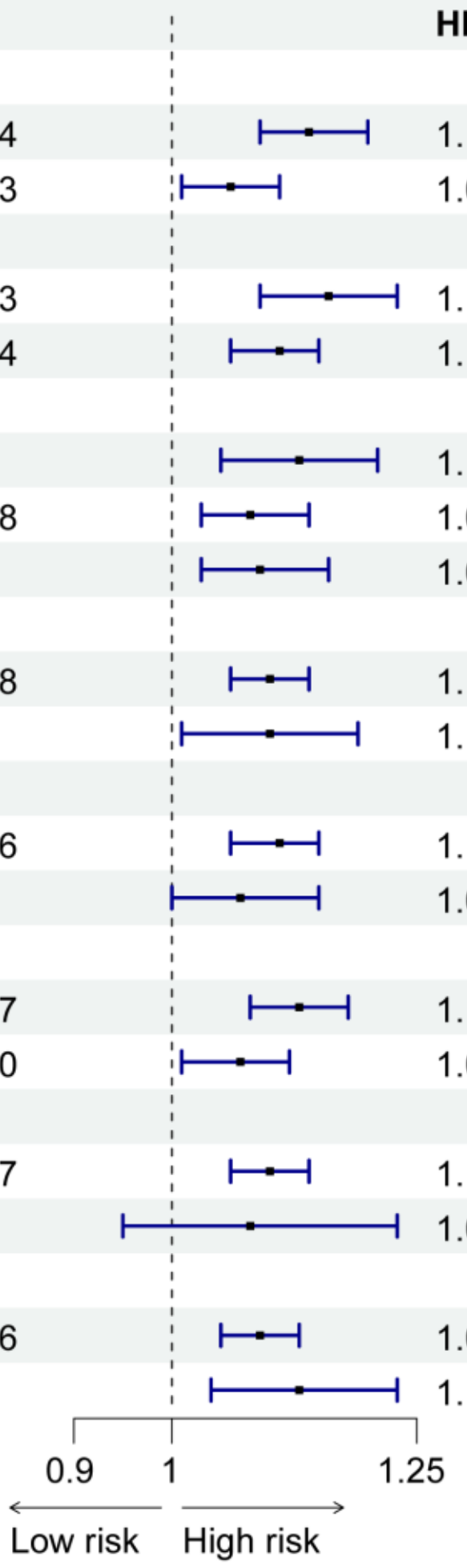

Supplementary Figure 5. Hazard ratios for pneumonia/influenza associated with appendectomy in subgroup analysis. Abbreviations: HR, hazard ratio; CI, confidence interval; P_i, *P* for interaction; BMI, body mass index; TDI, Townsend deprivation index; COPD, chronic obstructive pulmonary disease.

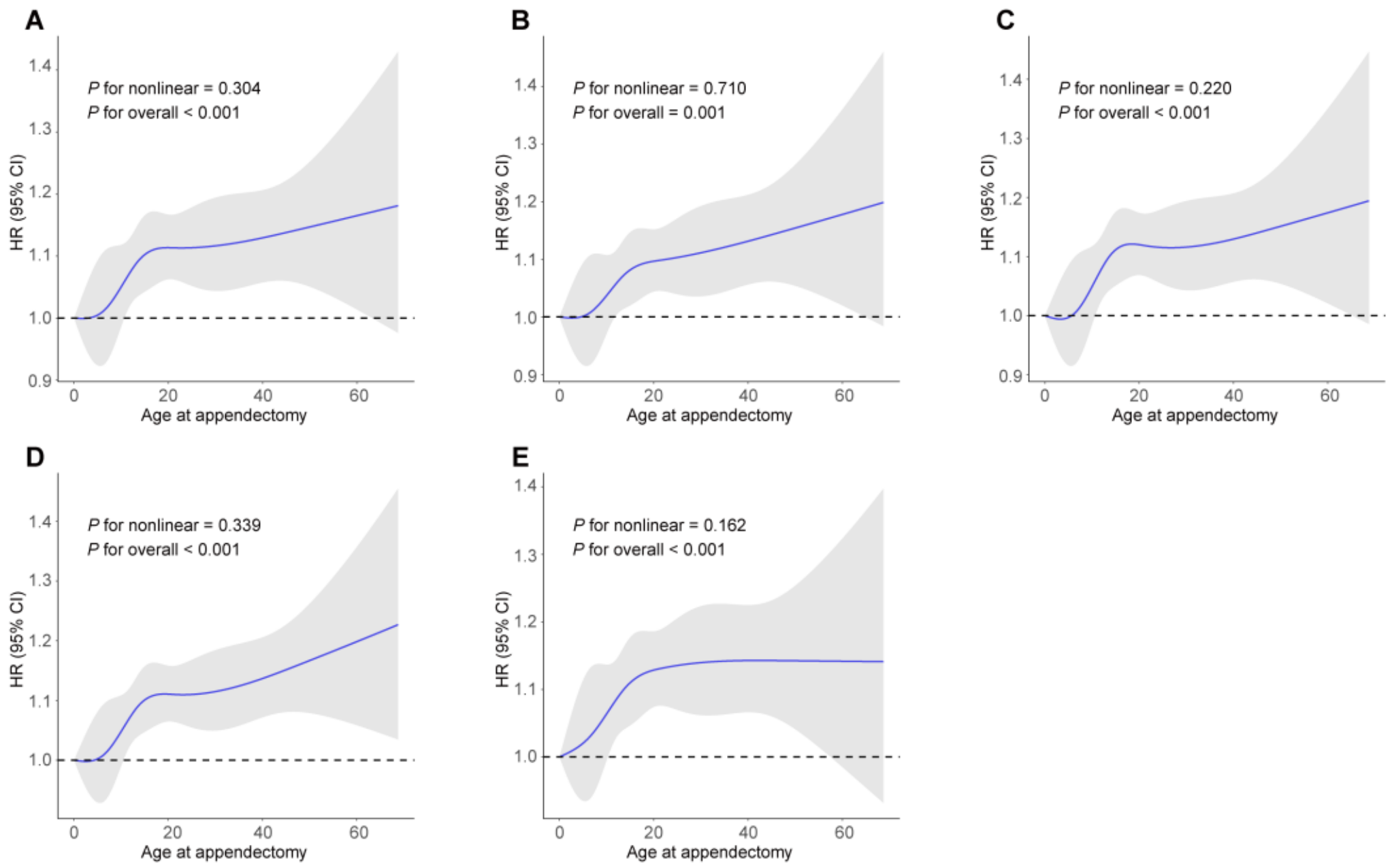

Supplementary Figure 6. Restricted cubic spline curves in sensitivity analysis. A. excluding self-reported pneumonia/influenza participants; B. excluding incident cases during the first two years of follow-up; C. excluding patients with a history of appendicitis at baseline; D. performing multiple imputation; E. limiting air pollution levels. Abbreviations: HR, hazard ratio; CI, confidence interval.

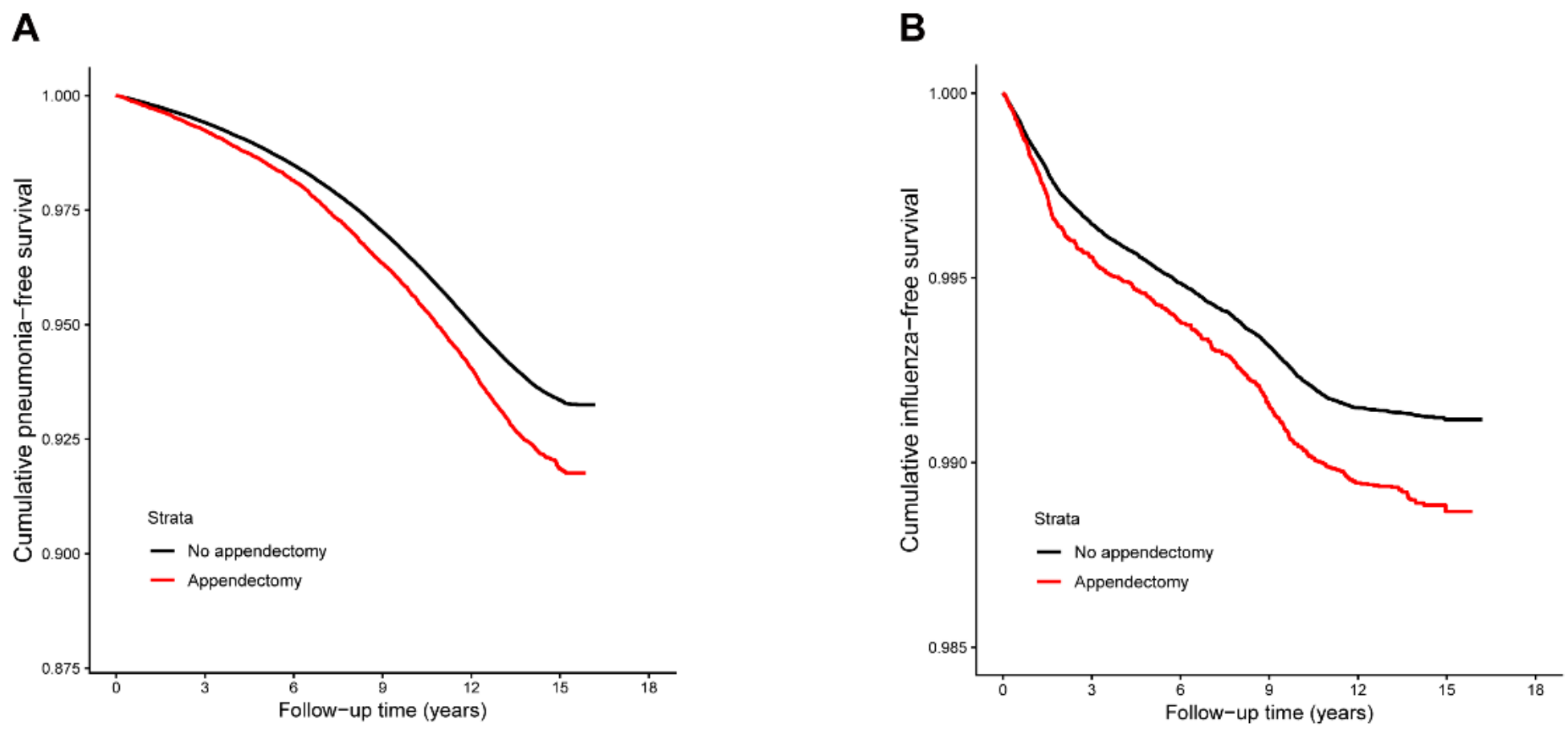

Supplementary Figure 7. Kaplan–Meier curves for component-specific outcomes according to appendectomy status. A. Pneumonia-free survival in participants with and without appendectomy. B. Influenza-free survival in participants with and without appendectomy.
